# Supplementary material for: Inbred Selection for Increased Resistance to Kernel Contamination with Fumonisins
Source: Toxins (Basel). 2023 Jul 4;15(7):444. doi: 10.3390/toxins15070444 (PMC10467120; doi:10.3390/toxins15070444)
Supplement: Supplementary file 1 [file toxins-15-00444-s001.zip › toxins-2462847-supplementary.pdf]

Supplementary Table S1. Means of parental inbreds for fumonisin content (mg/kg) and Fusarium ear rot (FER) score in a previous two-year evaluation under inoculation with *Fusarium verticillioides* [24].

Inbred	Kernel color	Kernel use	Kernel type	Heterotic group	Fumonisins	FER
**EP65**	White	Field corn	Flint	European	12.5	2.15
**F575**	White	Field corn	Flint	European	16.8	2.23
**EP31**	Yellow	Field corn	Flint	European	14.6	2.07
**EP39**	Yellow	Field corn	Flint	European	9.0	1.97
**A670**	Yellow	Field corn	Dent	Lancaster	14.7	2.00
**B93**	Yellow	Field corn	Dent	Lancaster	11.8	1.57
**H95**	Yellow	Field corn	Dent	Lancaster	6.0	2.07
**Oh43**	Yellow	Field corn	Dent	Lancaster	2.1	1.47
**A630**	Yellow	Field corn	Dent	Reid	30.3	2.28
**A654**	Yellow	Field corn	Dent	Reid	12.7	2.23
**A666**	Yellow	Field corn	Dent	Reid	10.0	1.96
